# Genome-wide association and selection studies for pod dehiscence resistance in the USDA soybean germplasm collection

**DOI:** 10.1371/journal.pone.0318815

**Published:** 2025-03-28

**Authors:** JaeBuhm Chun, Sadal Hwang

**Affiliations:** 1 National Institute of Crop Science, Crop Foundation Research Division, Iseo-myeon, Wanju-Gun, Jeonbuk-do, Republic of Korea; 2 United States of America Department of Agriculture, Agricultural Research Service, Sam Farr United States of America Crop Improvement and Protection Research Center, Salinas, California, United States of America; North Dakota State University, UNITED STATES OF AMERICA

## Abstract

As a domestication trait, pod dehiscence has a pleiotropic effect on agronomic traits and significantly contributes to yield loss in soybean. Population studies are still required to comprehend the genetic basis of dehiscence and to develop pod dehiscence-resistant cultivars with the optimal haplotype, thereby improving soybean production. We collected data for one wild (*Glycine soja*) (*G*. *soja*) and four cultivated (*Glycine max*) (*G*. *max*) populations from the USDA database. The *G*. *max* populations were evaluated in multi-environment conditions and used for genome-wide association study (GWAS) and selection. GWAS captured 86 quantitative trait loci (QTLs). Seventy-four new QTLs were colocalized in two different *G*. *max* populations, and 12 QTLs were closely mapped with previously reported QTLs. Eight out of 86 QTLs were associated with the domestication of pod dehiscence. We implemented marker-assisted selection (MAS) and genomic selection (GS) approaches to select pod dehiscence-resistant accessions with the best haplotype and lowest genomic breeding value (GBV), respectively. While our findings could be utilized for biology, genetics, and plant breeding, selecting pod dehiscence-resistant cultivars with the optimal haplotype will need further studies to confirm additional QTLs and assess advanced GS models.

## Introduction

Pod dehiscence is one of the key propagation steps in wild plant species [1]. As the pod matures and dries, a dehiscence zone forms in the suture between the lignified pod wall and replum, and dehiscence occurs due to pectin degradation and cell wall breakdown [2]. Pod dehiscence is a natural process that results in significant yield loss in crop production and impacts food security [3]. The regulation of pod dehiscence has evolved through artificial selection in many crop species, along with other domestication syndromes [4]. As one of the major crops in the USA, cultivated soybean (*G*. *max* L. Merr.) has been domesticated to be more resilient to pod dehiscence than its wild species (*G*. *soja* Sieb. and Zucc.) over several decades [5].

It is widely recognized that pod dehiscence in soybean is highly heritable. Some population studies measured pod dehiscence using the oven-dried method. The broad- and narrow-sense heritabilities for pod dehiscence ranged from 0.62 to 0.97 and 0.70 to 0.84, respectively, when pod dehiscence was assessed 7–20 days after maturity [6–7]. Other studies reported that the broad-sense heritability for pod dehiscence was over 0.8 when field evaluation was conducted 3–4 weeks after maturity [8–10]. These studies indicated that pod dehiscence variation was mainly attributed to genetic factor, regardless of pre- or post-harvest pod dehiscence [11].

Soybean has adapted to diverse climates and geographic conditions [12]. Some climate factors, such as precipitation and temperature, can significantly affect pod dehiscence. High temperature, rapid temperature change, high and continuous rainfall, and frequent transition between wet and dry conditions increased pod dehiscence [9]. The hot and humid weather in subtropical and tropical regions of Southeast Asia has posed challenges for soybean production due to pod dehiscence, as well as disease or insect infestation [13].

We now better understand the genetic loci controlling pod dehiscence in soybean. One restriction fragment length polymorphism (RFLP) marker, B122_1, was found on chromosome 16, and it explained 44% phenotypic variation in the Young × PI416937 recombinant inbred line (RIL) population [10]. One QTL was observed on chromosome 16 in the Toymusume × Hayahikari RIL population [14]. The QTL was located between two flanking simple sequence repeat (SSR) markers, Sat_366 and Sat_093. Four SSRs were identified on chromosomes 15 and 16 in the Tokei 780 × Hidaka 4 interspecific RIL population [15]. Eight SSRs were detected on chromosomes 2, 5, 9, 14, 16, 17, 18, 19, and 20 in the Keunolkong × Sinpaldalkong and Keunolkong × Iksan 10 RIL populations [16]. Fine mapping and genetics studies (e.g., gene expression analysis, immunocytochemistry, and complementation test) have confirmed two major QTLs, SHAT1-5 [17] and qPDH1 [18–19], on chromosome 16. From both QTLs, two genes, *SHAT1-5* [17] and *Pdh1* [20], were characterized and associated with the domestication of pod dehiscence. Both genes were involved in the lignification in the fiber cap cells of the ventral suture (*SHAT1*-*5*) and in the inner sclerenchyma of the pod wall (*Pdh1*). Excessive lignification enhanced pod dehiscence by reducing fiber in the suture and increasing twisting force on the pod wall. *NST1A* was identified on chromosome 7 [21]. It is orthologous to *NST1* in *Arabidopsis thaliana* (*A*. *thaliana*) and homologous to *SHAT1*-*5* in soybean. As a NAC domain transcription factor (TF), *NST1A* thickens the secondary cell wall.

Pod dehiscence is associated with major agronomic traits [9,22,23] and causes yield loss in soybean [3,24–26]. Developing pod dehiscence-resistant cultivars with the optimal haplotype through population studies is crucial for enhancing soybean production. Hence, our study aimed to 1) identify QTLs for pod dehiscence by GWAS, 2) detect QTLs for the domestication of pod dehiscence, and 3) select pod dehiscence-resistant accessions with the best haplotype and lowest GBV using MAS and GS.

## Materials and methods

### Public trait data

The pod dehiscence data of twenty-four *G*. *max* populations was collected in the germplasm resources information network (GRIN) (https://npgsweb.ars-grin.gov/gringlobal/descriptordetail?id=51012) (S1 Table). According to the GRIN, all field experiments were conducted using a 2- or 4-row plot over one or two years. At harvest, the two rows from the 2-row plot or the two center rows from the 4-row plot were assessed for pod dehiscence using a scale of 1 to 5 based on the percentage of open pods (e.g., 1 = 0%, 2 = 12.5%, 3 = 25%, 4 = 37.5%, and 5 ≥ 50%) [21,27–29]. Average values for pod dehiscence were only available for all accessions in 24 *G*. *max* populations, combining two replicates for one or two years. Using all trait data on a scale of 1 to 5 from 24 *G*. *max* populations, the 95% confidence interval (CI) of population standard deviation (*δ*) was estimated as follows: $\sqrt{\frac{(n-1)s^{2}}{\chi_{\alpha /2}^{2}}}<\delta <\sqrt{\frac{(n-1)s^{2}}{\chi_{1-\alpha /2}^{2}}}$ (*n*: total number of accessions of 24 *G. max* populations as a sample size, *s*^2^: sample variance of *n* accessions, *χ*^2^_α/2_ and *χ*^2^_1-α/2_: critical values with *n* – 1 degrees of freedom (*df*) in the chi-square (*χ*^2^) distribution, and α: 0.05 as type I error).

Four *G*. *max* populations, SOYBEAN.EVALUATION.1IL64 (1IL64), SOYBEAN.EVALUATION.1IL66 (1IL66), SOYBEAN.EVALUATION.MN0102 (MN0102), and SOYBEAN.EVALUATION.MS967 (MS967), were selected based on the upper limit value of the 95% CI of *δ.* Before subsequent data analyses, some accessions were excluded from each selected *G*. *max* population, considering the adaptation zones for soybean maturity groups (MGs) in the USA [30]. USDA reports described detailed information on field design, experiment, and evaluation in 1IL64 [27], 1IL66 [27], MN0102 [28], and MS967 [29]. 1IL64 and 1IL66 were planted in Illinois. MN0102 and MS967 were planted in Minnesota and Mississippi, respectively. Plots were 2 rows × 2.4 m long with 100 cm row spacing in 1IL64 and 1IL66. MN0102 and MS967 had 4 rows × 3.6 m long with 76 cm row spacing. Except for the accessions of 1IL64 (2 replicates per year), the accessions of 1IL66, MN0102, and MS967 were replicated once per year. Percentages of open pods in each *G*. *max* population were converted into arcsine-transformed values using the following equation: Y(x) = sin^-1^(square root of x) (Y(x): a transformed value and x: a percentage value / 100) [18,31]. The transformed average values were used as trait data for performing GWAS and GS.

### Public genotype data

The 50K single nucleotide polymorphism (SNP) data from the SoySNP50K iSelect BeadChip [32] has been developed to genotype the USDA soybean accessions. A high-quality dataset of 42,509 SNPs in HapMap format was obtained from SoyBase [33] (https://www.soybase.org/tools/snp50k/). Because 429 unanchored SNPs were excluded from the Williams 82 reference genome assembly V 2.0 (Wm82.a2.v1) [34], 42,080 SNPs remained for one *G*. *soja* and four *G*. *max* populations (1IL64, 1IL66, MN0102, and MS967). The *G*. *soja* population comprised 1,179 wild accessions (S2 Table) and was designated WS1179 in this study. Due to the lack of pod dehiscence data for wild accessions in the GRIN, WS1179 was used only for genotype data analyses.

After converting heterozygous genotypes into missing ones, SNPs with a high ratio of missing genotypes and monomorphism were processed in each population. The percentage of SNPs with missing genotypes exceeding 10% was 0.18%, 0.13%, 0.18%, 0.64%, and 4.58% in 1IL64, 1IL66, MN0102, MS967, and WS1179, respectively. These SNPs were removed to avoid potential biases in further data analyses. Additionally, 433 monomorphic SNPs with identical alleles across all five populations were discarded. A total of 39,509 SNPs were commonly present in all five populations. The linkage disequilibrium (LD) k-nearest neighbors algorithm [35] was employed to impute missing genotypes in TASSEL (V. 5.2.70) [36] with default parameter settings (number of SNPs in high LD = 30, number of neighbors (k) to use in imputation = 10, and maximum physical map distance between SNPs to search for LD = 10e^6^).

### Climate data

From the National Centers for Environmental Information (NCEI) (https://www.ncei.noaa.gov/access/monitoring/products/), we collected statewide average temperature and precipitation data from September to November, covering the years from 1895 to 2020 in the USA. The fall season from September to November was selected because soybean accessions with a wide range of MGs were harvested during this period in the USA [30]. Climate data was used to compare the Midwest (12 states) and the South (16 states) as the major divisions for soybean production.

### Putative domestication-related SNPs

Domestication-related SNPs were detected with three confirmation steps: 1) Tajima’s D [37], 2) *χ*^2^ goodness of fit test [38], and 3) Fixation index (F_ST_) [39].

1) Tajima’s D: Nucleotide diversity (*π)* [40], number of segregating sites (*θ*) [40]*,* and Tajima’s D values in 1IL64, 1IL66, MN0102, and MS967 were estimated using TASSEL (V. 5.2.70). A Tajima’s |D| value greater than 2 in a *G*. *max* population strongly indicated that the *G*. *max* population evolved under a non-neutral process and had domestication regions [41].
2) *χ*^2^ goodness of fit test: The test was performed using 39,509 common SNPs across all five populations. The observed frequencies of an allele from a SNP were calculated in WS1179, 1IL64, 1IL66, MN0102, and MS967. The expected frequencies of the allele from the SNP were calculated by dividing the sum of observed allele frequencies from WS1179 and a *G*. *max* population by 2. The *χ*^2^ goodness of fit test (*df* = 1) evaluated the difference in allele frequencies at a SNP between WS1179 and a *G*. *max* population using the following equation: *χ*^2^ = ∑ (observed allele frequency – expected allele frequency)^2^ / expected allele frequency. By employing the Benjamini–Hochberg procedure [42], the false discovery rate-adjusted *p*-value (*q*-value) was used as a criterion to find significant SNPs in one pair of *G*. *soja*-*G*. *max* dataset. Four *q*-values were generated in four pairs of *G*. *soja*-*G*. *max* datasets. SNPs with significance at one of the four *q*-values were considered domestication regions.
3) F_ST_: Like the *χ*^2^ goodness of fit test, the test was conducted using 39,509 common SNPs across all five populations. The F_ST_ value for a SNP was calculated using the following equation: F_ST_ =  *s*^2^ / (*T* × (1 – *T*)) (*s*^2^: sample variance in the frequencies of an allele between two different populations and *T*: average frequency of the allele in the total population). As a measure of population differentiation due to genetic structure, the range of F_ST_ value was defined to indicate moderate differentiation (0.05–0.15), high differentiation (0.15–0.25), and substantial differentiation ( > 0.25) [43]. SNPs with F_ST_ values greater than 0.05 between WS1179 and a *G*. *max* population were considered domestication regions.

### Gene ontology analysis

Domestication-related SNPs were used for the gene ontology (GO) analysis. SNPs located within soybean genes with the Glyma2.0 gene model were selected. SoyBase [33] (https://www.soybase.org/tools/analysis/go.html) and the GO database (http://www.geneontology.org/) [44] were used to search for GO terms.

### Population structure, principal component, and linkage disequilibrium analyses

The K values of WS1179, 1IL64, 1IL66, MN0102, and MS967 were inferred by Structure (V. 2.3.4) [45]. In each population, 10K SNPs were used to reduce computation. The top 500 SNPs with the highest minor allele frequency (MAF) were selected and well-distributed on each chromosome (500 SNPs ×  20 chromosomes =  10K SNPs). The genotype data of each population was converted into numerical data (AA = 1, TT = 2, GG = 3, and CC = 4). The length of the Burnin period was 10,000, and the number of Markov Chain Monte Carlo replications after the Burnin was 10,000. Correlated allele frequency [45] and admixture [46] were used as variables in the statistical model without prior population knowledge. It was assumed that the value of K ranged from 1 to 30. The statistical test was iterated 20 times for each K value. When a K value was input, all hyperparameters, allele frequency (*λ*), degree of admixture (*α*), and F_ST_, were estimated by Bayesian estimation in each statistical test. Twenty log-likelihood values were obtained, and their sample standard deviation (*s*) was calculated. The estimation of ∆ K [47] was as follows: ∆K = L″(*λ*, *α*, F_ST_|K) / *s* L(*λ*, *α*, F_ST_|K) (L″(*λ*, *α*, F_ST_|K): second-order rate of change in average log-likelihood values between K – 1 and K and *s* L(*λ*, *α*, F_ST_|K): *s* of 20 log-likelihood values at K). Average log-likelihood value [48] and ΔK were used to determine the best K for each population. Additionally, the Q matrix of each *G*. *max* population was created for GWAS. A Q matrix provided information about how accessions were clustered in K.

Principal component analysis (PCA) was conducted by TASSEL (V. 5.2.70) using 39,509 common SNPs across all five populations. Before performing PCA, the genotype data of each population was converted into numerical data (homozygous major allele = 1 and homozygous minor allele = 0).

LD was assessed in each *G*. *max* population. Monomorphic SNPs were excluded from each *G*. *max* population. TASSEL (V. 5.2.70) calculated the squared correlation coefficient (*r*^2^) [49] values between any two SNPs within a 30kb window. A *r*^2^ value greater than 0.8 indicated a strong association between alleles, signifying high LD.

### Genome-wide association study

GWAS was performed to identify QTLs for pod dehiscence in each *G*. *max* population. The k-nearest neighbors algorithm [50] was employed to impute a few missing trait values in TASSEL (V. 5.2.70) with default parameter settings (number of neighbors (k) to use in imputation = 5 and distance measures for computing nearest neighbors = Euclidean method). SNPs with MAF less than 5% and monomorphism were filtered out. An identical by descent (IBD)-based kinship (*k*) matrix [51] was created in TASSEL (V. 5.2.70).

Trait, genotype, and kinship data files were uploaded to GAPIT (V. 3.0) [52] in R (V. 4.2.1) [53]. In the GAPIT() function, the HapMap genotype data of each *G*. *max* population was converted into numerical data (homozygous major allele = 2 and homozygous minor allele = 0). Using the “Model.selection = TRUE” argument, forward model selection with the Bayesian information criterion determined the optimal number of PCs and created a PC matrix. In addition, another type of marker-based *k* matrix [54] was generated. Six statistical GWAS models, general linear model [55], mixed linear model [56], SUPER [57], MLMM [58], FarmCPU [59], and BLINK [60], were run simultaneously. Except for BLINK, Q, PC, and *k* matrices were utilized as cofactors in five GWAS models. A Q matrix was used interchangeably with a PC matrix. Likewise, two IBD- and marker-based *k* matrices were also interchangeable. A *q*-value was used to declare the rejection of the null hypothesis (H_0_: No QTL for a tested SNP).

### QTL data mining and confirmation

We examined previously reported QTLs that were tightly linked to those identified in this study. The relationship between the Consensus 4.0 genetic map [61] and the physical map [34] indicated that 5 cM on the Consensus 4.0 genetic map was equivalent to 2.2 Mb on the physical map. A distance of 5 cM or less was defined as a region of tight linkage. Therefore, QTLs for pod dehiscence identified in former studies were collected within a 2.2 Mb window of the QTLs discovered in this study.

We gathered data on the verified gene locations from QTLs and the CIs of QTL positions. In this study, the *p*-values for SNPs tested in each GWAS model were converted into logarithm of odds (LOD) scores using the following equation: LOD score = log_10_(*p*-value^-1^). The 95% CIs of QTL positions were calculated using the 1.5-LOD support interval [62–63]. On the other hand, the gene locations of major QTLs were acquired from previous gene characterization studies. Since the QTLs obtained from previous RIL population studies provided limited information about the CIs of QTL positions, we collected information about QTL marker positions, R^2^ values, and population sizes from these studies. Then, the 95% CIs of QTL positions were estimated using the following equation: 95% CI for a RIL population = 163 / (N ×  R^2^) (R^2^: phenotypic variance explained by a QTL and N: population size) [64]. The positions and 95% CIs of all QTLs were projected onto the Consensus 4.0 genetic map for QTL confirmation [65]. Due to the framework consisting of various markers (e.g., SNP, SSR, RFLP, and morphological markers), the Consensus 4.0 genetic map has been utilized to confirm the CIs of QTL positions across different population studies.

### Candidate gene and sequence analyses

The candidate genes for QTLs were screened within the 95% CIs of QTL positions using the 1.5-LOD support interval [62–63]. The sequences of soybean candidate genes with the Glyma2.0 gene model in the Wm82.a2.v1 were available from SoyBase [33] (https://www.soybase.org/tools/browsers/). The *Arabidopsis* information resource (TAIR) BLASTN (V. 2.9.0+) (https://www.arabidopsis.org/tools/blast/) was used to identify *A*. *thaliana* genes homologous to soybean candidate genes using two criteria, Bit score ( > 50) and E-value ( < 0.05).

### Marker-assisted selection

MAS was performed using 86 QTLs that were identified by GWAS. The favorable and unfavorable alleles of a SNP in each *G*. *max* population were determined by comparing the average trait values of two accession groups based on the genotypes of alleles. The allele from the accession group with a lower average trait value was defined as a favorable allele. The percentage of 86 favorable alleles was calculated in an accession group with a trait value of *j* (*j* = 1, 2, 3, 4, and 5 on a scale of 1 to 5). The selection efficiency of 86 SNPs in each *G*. *max* population was defined as follows: Selection efficiency of 86 SNPs =  (the average percentage of 86 favorable alleles in the accession group with a trait value of 1 + the average percentage of 86 unfavorable alleles in the accession group with a trait value of 5) / 2 / 100. The upper extreme trait value was 4 in MS967.

At a given SNP in each *G*. *max* population, the percentage of a favorable allele in an accession group with a trait value of *j* (*j* = 1, 2, 3, 4, and 5 on a scale of 1 to 5) was calculated as follows: The percentage of a favorable allele in an accession group with a trait value of *j* = (number of accessions with a favorable allele in an accession group with a trait value of *j*) / (total number of accessions in an accession group with a trait value of *j*) x 100. The selection efficiency of a SNP in each *G*. *max* population was defined as follows: Selection efficiency of a SNP = (the percentage of a favorable allele in the accession group with a trait value of 1 + the percentage of an unfavorable allele in the accession group with a trait value of 5) / 2 / 100.

### Genomic selection

The trait, genotype, and PC matrix data for GWAS were also used for GS in each *G*. *max* population. Three basic models for the best linear unbiased prediction (BLUP), genomic best linear unbiased prediction (gBLUP) [66], compressed gBLUP (cBLUP) [67], and SUPER gBLUP (sBLUP) [67], were implemented for GS in GAPIT (V. 3.0). To investigate genomic prediction accuracy, *K*-fold cross-validation with replacement was employed at different folds (2, 5, 10, and 20) in gBLUP. The compressed accession groups were assigned as reference or inference panels. As the indicator of genomic prediction accuracy, the average correlation coefficient (*r*) [68] values between trait value and genomic prediction value at different folds were estimated with 1,000 times repetitions. The average *r* value ( ≥ 0.8) across the different folds was utilized as the criterion for selecting a *G*. *max* population with high genomic prediction accuracy. The *r* value between trait value and GBV in the selected *G*. *max* population was estimated in each BLUP model to measure selection efficiency.

### Statistical analysis

In SAS 9.4 (SAS Institute Inc., Cary, NC), PROC TTEST, PROC FREQ, PROC GLM, PROC CORR, and PROC UNIVARIATE statements were used for *t*, *χ*^2^, analysis of variance (ANOVA), correlation, and normality tests, respectively. The PROC TTEST statement was used to analyze climate data. The Welch’s *t*-test [69] was applied as an independent *t*-test, assuming two division groups had different sample sizes and unequal variances. The Welch-Satterthwaite equation [69] was employed to estimate the *df* from a linear combination of the two group variances. As part of the analysis for domestication-related SNPs, the PROC FREQ statement was utilized to calculate the observed frequencies of an allele at a SNP between *G*. *soja* and *G*. *max* populations. The CHISQ option estimated the expected frequencies of the allele. Additionally, the CHISQ option was used to conduct the *χ*^2^ goodness of fit test [38] using the observed and expected allele frequencies. The PROC GLM statement was employed for ANOVA in MAS and GS analyses. In one- or two-way ANOVA, all independent variables had fixed effects. As a *post hoc* test, the Waller-Duncan k-ratio *t*-test [70] was conducted for multiple comparison tests (MCTs) if one-way ANOVA yielded a significant result. In the PROC CORR statement, the Pearson *r* [68] was used by default to measure the relationship between two variables. In the PROC UNIVARIATE statement, the NORMAL option was used to assess whether datasets, including a transformed pod dehiscence dataset, followed a normal distribution in the linear models of *t*-test, ANOVA, and GWAS. The Shapiro-Wilk test was employed to determine if a dataset was normally distributed. A one-tailed test with a 5% alpha level was applied for all statistical significance tests in this study.

## Results

### Selection of *G*. *max* populations

Pod dehiscence was evaluated in twenty-four *G*. *max* populations (S1 Table). Using all trait data from 24 *G*. *max* populations, the 95% CI for *δ* ranged from 0.587 to 0.698 in the *χ*^2^ distribution. Four *G*. *max* populations, 1IL64, 1IL66, MN0102, and MS967, were selected (Table 1). The sample standard deviations of the selected *G*. *max* populations exceeded the upper limit of the 95% CI of *δ*, implying a high likelihood of identifying more QTLs due to greater variation. The MGs of 1IL64, 1IL66, and MN0102 ranged from 0 to IV. The MGs of MS967 ranged from V to VIII.

**Table 1:** Information about four *G*. *max* populations for pod dehiscence study.

Population^°^	Population^†^	Location^¶^	Year^§^	MG^‡^	Accession^£^	Range^¥^	*s* ^*^
SOYBEAN.EVALUATION.1IL64	1IL64	IL	1964	I-II	721	1–5	1.12
SOYBEAN.EVALUATION.1IL66	1IL66	IL	1965 – 1966	III-IV	1,140	1–5	1.07
SOYBEAN.EVALUATION.MN0102	MN0102	MN	2001 – 2002	0-I	485	1–5	1.19
SOYBEAN.EVALUATION.MS967	MS967	MS	1996 – 1997	V-VIII	1,109	1–4	0.70

° Population name in the GRIN.

† Replaced population name.

¶ As field experiment sites, IL, MN, and MS indicated Illinois, Minnesota, and Mississippi, respectively.

§ Field test year.

‡ Maturity group.

£ Number of accessions. Some accessions in each *G*. *max* population were discarded from the original population based on the soybean maturity zone in the USA [30].

¥ Range of trait value on a scale of 1 to 5. The upper extreme trait value was 4 in MS967.

* The *s* of each *G*. *max* population was recalculated due to a change in the number of accessions.

### Evaluation of two climate factors

The soybean maturity zone in the USA indicated that three *G*. *max* populations (1IL64, 1IL66, and MN0102) and MS967 have been adapted in the Midwest and South, respectively (S1 Fig). Two major soybean production divisions, the Midwest and South, were compared for differences in temperature and precipitation (S1 Fig). According to the *t*-tests, the South showed higher temperature and precipitation than the Midwest (*p*-values < 1e-03). The average temperature and precipitation differences between the Midwest and South were 33.4 °F and 45.7 mm, respectively. The average temperature and precipitation in the South were 108.8 °F and 92.3 mm, respectively.

### Putative domestication-related SNPs

The values of *π*, *θ*, and Tajima’s D were calculated for each *G*. *max* population. The values of *π* and *θ* ranged from 0.279 to 0.294 and 0.121 to 0.139 per SNP, respectively. Tajima’s D values ranged from 3.31 to 4.06, implying that all *G*. *max* populations lacked rare alleles and evolved under a non-random process.

As the criteria for the *χ*^2^ goodness of fit test between WS1179 and a *G*. *max* population, four *q*-values, 0.0038, 0.0035, 0.0038, and 0.0029, were used to find significant SNPs in four pairs of datasets, WS1179-1IL64, WS1179-1IL66, WS1179-MN0102, and WS1179-MS967, respectively (S3 Table and S2 Fig). A total of 4,280 SNPs were significant at one of the four *q*-values. In 4,280 SNPs, 2,204 SNPs were significant in all *G*. *max* populations. The rest of the 2,076 SNPs were population-specifically significant in 1IL64 (284 SNPs), 1IL66 (173 SNPs), MN0102 (292 SNPs), and MS967 (1,327 SNPs).

**Fig 1:**
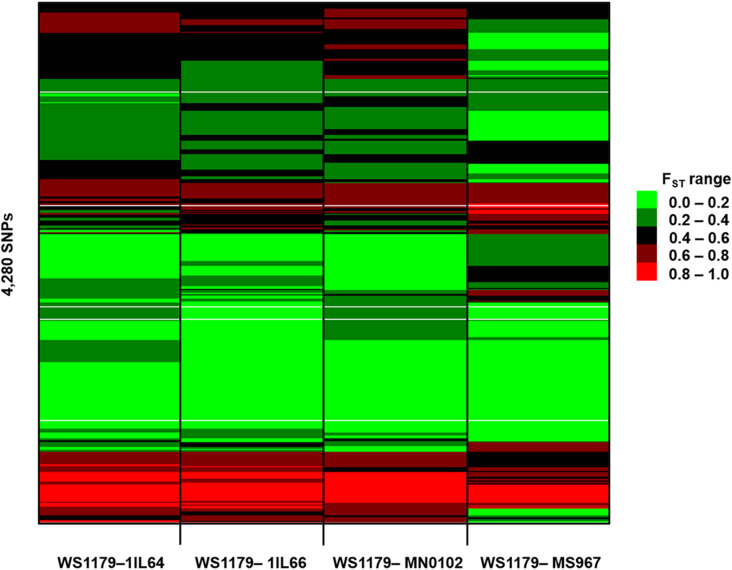
F_ST_ values of putative domestication-related SNPs between WS1179 and a *G*. *max* population. The F_ST_ values for 4,280 SNPs were estimated in four pairs of datasets, WS1179-1IL64, WS1179-1IL66, WS1179-MN0102, and WS1179-MS967.

The F_ST_ values for 4,280 SNPs were estimated between WS1179 and a *G*. *max* population (S3 Table and Fig 1). SNPs with significant *q*-values in the *χ*^2^ goodness of fit test had F_ST_ values greater than 0.05. The average F_ST_ values were 0.38, 0.36, 0.38, and 0.33 in four pairs of datasets, WS1179-1IL64, WS1179-1IL66, WS1179-MN0102, and WS1179-MS967, respectively. These average F_ST_ values demonstrated that the genetic structure between *G*. *soja* and *G*. *max* populations was substantially different. The average F_ST_ values were estimated among *G*. *max* populations. The average F_ST_ values among 1IL64, 1IL66, and MN0102 ranged from 0.01 to 0.02, indicating high genetic similarity among the populations. The average F_ST_ values between MS967 and the other three *G*. *max* populations ranged from 0.04 to 0.06, showing that MS967 was moderately differentiated from 1IL64, 1IL66, and MN0102. Considering Tajima’s D, *χ*^2^ goodness of fit test, and F_ST_, 4,280 SNPs were detected as putative domestication-related SNPs.

### Gene ontology

A total of 1,515 out of 4,280 SNPs were located within soybean genes with the Glyma2.0 gene model (S3 Table). Therefore, 1,515 putative domestication-related genes were used for GO analysis. Based on the biological process GO term, 29 GO terms were identified (S3 Fig). Except for the GO term, uncategorized (no GO ID), the top 5 GO terms, signal transduction (GO:0007165) (12.03%), transport (GO:0006810) (9.49%), multicellular organismal development (GO:0007275) (7.91%), flower development (GO:0009908) (6.33%), and cell differentiation (GO:0030154) (5.85%), were annotated in the largest number of genes.

### Population structure and linkage disequilibrium

**Fig 2:**
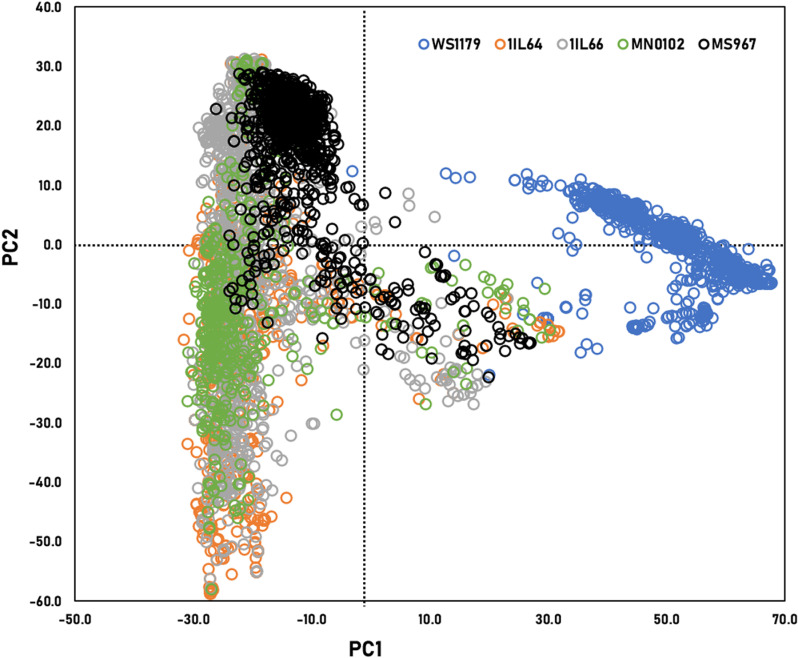
PCA using 39,509 common SNPs across all five populations. Light blue, orange, gray, green, and black circles represented the accessions of WS1179, 1IL64, 1IL66, MN0102, and MS967, respectively.

The accessions of WS1179 originated from seven countries: China, Japan, South Korea, the Philippines, Russia, Taiwan, and the USA (S2 Table). WS1179 had eight subpopulations (K = 8). The K values of 1IL64, 1IL66, MN0102, and MS967 were 25, 23, 21, and 22, respectively (S4 Fig). PCA was performed to investigate the clustering of five populations in a two-dimensional coordinate system using principal component 1 (PC1) and principal component 2 (PC2) (Fig 2). The eigenvalues for PC1 and PC2 were 998.04 and 398.91. The percentage of variance explained by PC1 was 72%. The average coordinates were (21.5, ‒10.2), (0.1, ‒13.8), (-3.5, ‒13.3), (‒0.3, ‒13.5), and (0.5, 3.2) in WS1179, 1IL64, 1IL66, MN0102, and MS967, respectively. WS1179 was clustered separately from all *G*. *max* populations. MS967 showed a slightly different grouping compared to the other three *G*. *max* populations. In LD, the average *r*^2^ values of 1IL64, 1IL66, MN0102, and MS967 were 0.35, 0.31, 0.33, and 0.26, respectively.

### QTL analysis

Most QTLs for pod dehiscence were initially identified in each *G*. *max* population using PC and marker-based *k* matrices in GWAS models (S5 Fig). When we employed different combinations of cofactors in the GWAS models, additional QTLs were also discovered. Nevertheless, the overall QTL findings for each combination of cofactors remained largely consistent.

**Fig 3:**
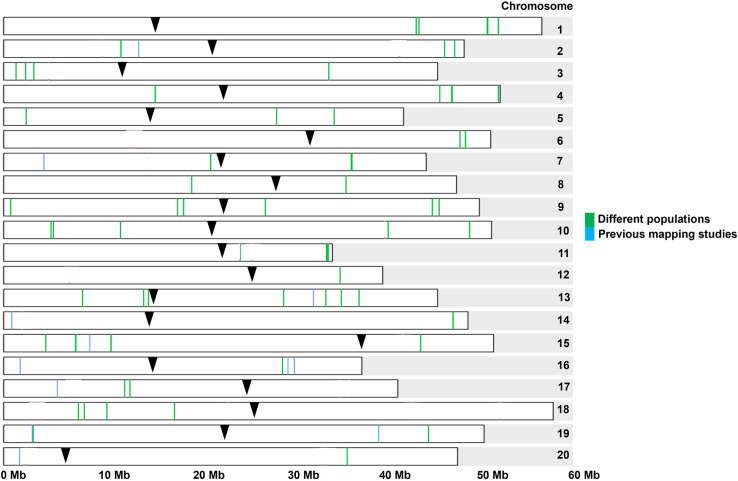
QTLs identified by GWAS in four *G*. *max* populations. The black inverted triangle indicated the centromere of each chromosome. The green vertical lines displayed 74 QTLs identified in two different *G*. *max* populations. The blue vertical lines showed 12 QTLs located near previously reported QTLs within a 2.2 Mb window.

We chose QTLs that met one of two criteria: 1) QTLs colocalized in different *G*. *max* populations, or 2) QTLs tightly linked to previously reported QTLs. Eighty-six QTLs (S4 Table and Fig 3) were ultimately determined. Seventy-four new QTLs were colocalized in two different *G*. *max* populations. Twelve QTLs identified in a single *G*. *max* population were located near previously reported QTLs within a 2.2 Mb window. *NST1A* [21], *SHAT1-5* [17], and *Pdh1* [20] collected from previous gene characterization studies were closely located to 3 QTLs, ss715598070, ss715623567, and ss715624201, respectively. *NST1A* was located 93 kb upstream from ss715598070 on chromosome 7. *SHAT1-5* was located 283 bp upstream from ss715623567 on chromosome 16. *Pdh1* was located 42 kb downstream from ss715624201 on chromosome 16. *Pdh1* was located between ss715624199 and ss715624201. ss715624199 was polymorphic in all *G*. *max* populations and located 20 kb downstream from *Pdh1*. Both ss715624199 and ss715624201 showed high LD across the *G*. *max* populations (average *r*^2^ = 0.85). One RFLP [10] and eight SSRs [15–16] collected from previous RIL population studies (S5 Table) were located within a range of 8 kb to 1.8 Mb from 9 QTLs (ss715581242, ss715615326, ss715620013, ss715623192, ss715624318, ss715628052, ss715633661, ss715634837, and ss715636770) on chromosomes 2, 13, 14, 15, 16, 17, 19, and 20.

The *r*^2^ values of 9 pairs of QTLs (ss715579431-ss715579443, ss715580084-ss715580088, ss715610565-ss715610559, ss715610541-ss715610538, ss715621953-ss715621956, ss715620316-ss715620318, ss715624103-ss715624106, ss715625940-ss715625948, and ss715633661-ss715633692) on chromosomes 1, 11, 15, 16, 17, and 19 were greater than 0.8 in all *G*. *max* populations. Eight QTL, ss715579431, ss715583442, ss715588460, ss715598070, ss715605375, ss715607935, ss715610541, and ss715624201, were observed as domestication-related SNPs on chromosomes 1, 2, 4, 7, 9, 10, 11, and 16, respectively.

According to the 95% CIs of 86 QTL positions (S4 Table), soybean candidate genes from 86 QTLs and their homologous *A*. *thaliana* genes were described in S6 Table. Seventy-four new QTLs were confirmed in two different *G*. *max* populations over multiple years. In four pairs of QTLs (ss715580083-ss715580084, ss715588460-ss715588468, ss715610541-ss715610538, and ss715621953- ss715621956) on chromosomes 1, 4, 11, and 15, the 95% CIs of each pair of QTL positions overlapped. It was presumed that each pair of QTLs represented the same QTL. Because 12 out of 86 QTLs were identified in a single *G*. *max* population, we needed confirmation. As two major soybean genes related to pod dehiscence, Glyma.16G019400 (*SHAT1***-***5*) and Glyma.16G141500 (*Pdh1*) on chromosome 16 were located within the 95% CIs of two QTL positions for ss715623567 and ss715624201, respectively (S6 Table). Glyma.07G050600 (*NST1A*) on chromosome 7 was homologous to Glyma.16G019400 (*SHAT1*-*5*) in soybean but was not located within the 95% CI of the QTL position for ss715598070. The 95% CIs of QTL positions for one RFLP and eight SSRs (S5 Table) overlapped with the 95% CIs of 9 QTL positions (S4 Table).

### Marker-assisted selection

**Fig 4:**
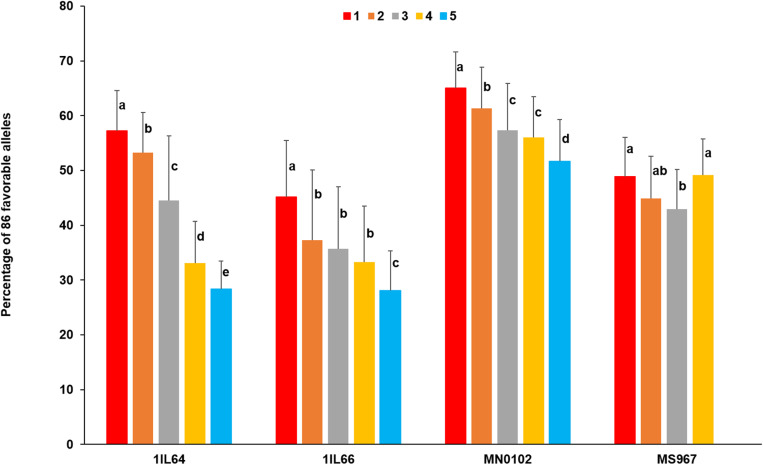
Percentage of 86 favorable alleles in accession groups based on trait values in each *G*. *max* population. Red, orange, gray, golden yellow, and blue rectangular bars represented accession groups with trait values of 1, 2, 3, 4, and 5, respectively. The upper extreme trait value was 4 in MS967. The Waller-Duncan k-ratio *t*-test was conducted for MCTs in each *G*. *max* population. The critical values for MCTs were 1.73, 1.74, 1.75, and 1.76 in 1IL64, 1IL66, MN0102, and MS967, respectively.

The favorable alleles of 86 QTLs were identified in each *G*. *max* population (S7 Table). The percentage of 86 favorable alleles was calculated in accession groups based on trait values in each *G*. *max* population (Fig 4). Two-way ANOVA indicated that accession group, population, and their interaction effects were highly significant (*p*-values < 1e-03) across the *G*. *max* populations. In each *G*. *max* population, one-way ANOVA showed that the percentage of 86 favorable alleles among accession groups was significantly different (*p*-values < 1e-03).

Using two accession groups with extreme trait values, the selection efficiencies of 86 SNPs were 0.65, 0.59, 0.57, and 0.50 in 1IL64, 1IL66, MN0102, and MS967, respectively. The average selection efficiency of 86 SNPs was 0.57 across the *G*. *max* populations. The accession with the best combination of 86 favorable alleles was identified in each *G*. *max* population. Four accessions, PI89059 (72.1% / 62 QTLs), PI153292 (66.3% / 57 QTLs), PI592962B (76.7% / 66 QTLs), and PI416873B (66.3% / 57 QTLs), were found in 1IL64, 1IL66, MN0102, and MS967, respectively. The trait values of the four accessions were 1, indicating resistance to pod dehiscence.

The selection efficiency of each SNP was estimated. Table 2 displayed the top 5 SNPs with excellent selection efficiency in each *G*. *max* population. The SNP, ss715624201, was ranked first or second in 1IL64, 1IL66, and MN0102. The selection efficiency of ss715624201 ranged from 0.78 to 0.97. Two SNPs, ss715591071 and ss715606969, were ranked first in 1IL66 and MS967. Two SNPs, ss715623567 and ss715616944, were commonly observed in 1IL64 and 1IL66. The selection efficiencies of ss715623567 were 0.90 and 0.72 in 1IL64 and 1IL66. In MN0102, the selection efficiency of ss715598070 was 0.71. As previously reported QTLs (S4 Table), three SNPs, ss715598070, ss715623567, and ss715624201, were closely located to *NST1A*, *SHAT1*-*5*, and *Pdh1*, respectively.

**Table 2:** Top 5 SNPs with the highest selection efficiency in each *G*. *max* population.

Population	Rank	Chromosome	SNP	Allele^¥^	Favorable allele^‡^	Selection efficiency
1IL64	1	16	ss715624201	T/C	T	0.97
	2	16	ss715623567	C/T	C	0.90
	3	12	ss715612572	A/G	A	0.89
	4	13	ss715616944	T/G	T	0.87
	5	7	ss715596809	G/A	G	0.83
1IL66	1	5	ss715591071	T/C	T	0.82
	2	16	ss715624201	T/C	T	0.78
	3	16	ss715623567	C/T	C	0.72
	4	13	ss715616944	G/T	T	0.72
	5	9	ss715604557	C/T	C	0.72
MN0102	1	16	ss715624201	T/C	T	0.79
	2	20	ss715636941	A/G	A	0.78
	3	4	ss715588460	C/A	C	0.72
	4	3	ss715584417	A/C	A	0.72
	5	7	ss715598070	A/C	A	0.71
MS967	1	10	ss715606969	C/T	C	0.72
	2	20	ss715637668	G/T	G	0.71
	3	19	ss715635382	A/G	G	0.71
	4	10	ss715608238	A/C	C	0.71
	5	18	ss715632696	G/A	G	0.71

¥ Biallelic variants of each SNP.

‡ Allele contributed to the decrease in the number of open pods at harvest.

### Genomic selection

**Fig 5:**
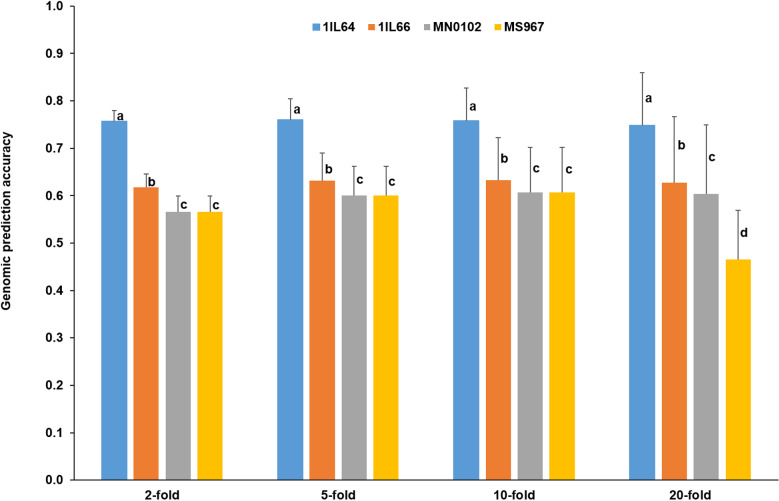
Genomic prediction accuracies of four *G*. *max* populations at different fold levels in gBLUP. Blue, orange, gray, and golden yellow rectangular bars represented 1IL64, 1IL66, MN0102, and MS967, respectively. The Waller-Duncan k-ratio *t*-test was conducted for MCTs at each fold level. The critical values for MCTs were 1.72144, 1.72146, 1.72144, and 1.72143 at 2-, 5-, 10-, and 20-fold levels, respectively.

The genomic prediction accuracies of four *G*. *max* populations were evaluated at different fold levels (Fig 5). Two-way ANOVA indicated that population, fold level, and their interaction effects were highly significant (*p*-values < 1e-03) across the fold levels. At each fold level, one-way ANOVA showed that the genomic prediction accuracies of four *G*. *max* populations were significantly different (*p*-values < 1e-03). The average genomic prediction accuracies across the fold levels were 0.76, 0.63, 0.59, and 0.56 in 1IL64, 1IL66, MN0102, and MS967, respectively.

Because the average genomic prediction accuracy of 1IL64 was approximately 0.8, 1IL64 was selected for GS. The *r* values between trait value and GBV in 1IL64 were 0.148 (*p*-value < 1e-04), 0.153 (*p*-value < 1e-04), and 0.403 (*p*-value < 1e-04) in gBLUP, cBLUP, and sBLUP, respectively. 1IL64 had the largest *r* value in sBLUP, indicating the highest selection efficiency.

**Fig 6:**
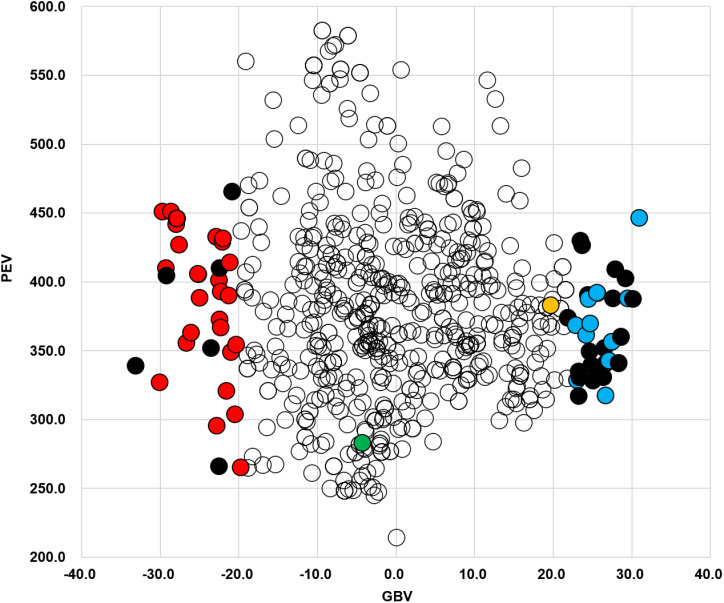
Distribution of the GBV and PEV for accessions in 1IL64 when sBLUP was applied. In the bottom and top 5% of the GBV distribution, solid red, blue, and black circles represented accessions with trait values of 1, 5, and others, respectively. Solid green and orange circles corresponded to two accessions, PI89059 and PI181532, respectively.

sBLUP was employed to estimate the GBV and prediction error variance (PEV) for accessions in 1IL64 (S8 Table and Fig 6). The GBV and PEV ranged from -33.06 to 31.03 and 214.23 to 582.37, respectively. The top 10 pod dehiscence-resistant accessions, PI84666-1, PI171421, PI88797, PI73780, PI153315, PI92470, PI142491, PI68696, PI79761, and PI88805-4, showed the lowest GBV. Except for PI84666-1 and PI153315, the trait values of eight accessions were 1. On the other hand, PI181570, PI90575, PI548329, PI84896, PI196158, PI181537, PI81033, PI189962, PI229354, and PI181548 were selected as the top 10 pod dehiscence-susceptible accessions with the highest GBV. The trait values of only two accessions, PI181570 and PI548329, were 5.

Two groups of 36 accessions were evaluated in the bottom and top 5% of the GBV distribution (Fig 6). The trait values of 30 (83.3%) and 13 (36.1%) accessions were 1 and 5 in the bottom and top 5%. In MAS, PI89059 showed the highest percentage (72.1%) of 86 favorable alleles with a trait value of 1. In contrast, PI181532 had the highest percentage (80.2%) of 86 unfavorable alleles with a trait value of 5. The GBV (19.7) of PI181532 was near the top 5%, while the GBV (-4.23) of PI89059 was in the middle of the distribution.

## Discussion

Soybean candidate genes from 86 QTLs and their homologous *A*. *thaliana* genes were listed in S6 Table. Based on previous *A*. *thaliana* studies, we highlighted some notable *A*. *thaliana* genes involved in the lignification, breakdown, and biosynthesis of the cell wall, serving as critical processes for pod dehiscence.

We first explored *A*. *thaliana* genes related to cell wall components. The mature sclerenchyma of the pod wall consists of dead cells. AT5G01660.1 [71] functioned as a protein with the development and cell death (DCD) domain and was homologous to Glyma.01G126500 on chromosome 1. Laccase synthesizes lignin as the multicopper oxidase of phenols [72]. As one of the 17 laccase family genes, AT5G03260.1 (laccase 11) [73] played a role in lignin biosynthesis and was homologous to Glyma.01G173600 on chromosome 1. As an auxin response factor (ARF), AT5G62000.1 (ARF2) [74] mutation study indicated that *arf2* allele had a pleiotropic effect on multiple traits, including delayed silique dehiscence. AT5G62000.1 was homologous to Glyma.04G200600 on chromosome 4. Hydrolase and cellulose synthase (CESA) are essential enzymes forming the cell wall. AT1G32860.1 [75] hydrolyzed the glycosidic bond between two or more carbohydrates, modifying the cell wall during silique dehiscence. AT5G17420.1 (CESA7) [76] mutant study showed that *cesa7* affected cell growth, cell wall integrity, and cellulose level. AT1G32860.1 and AT5G17420.1 were homologous to Glyma.10G172300 and Glyma.17G072200 on chromosomes 10 and 17, respectively. Pectin is a complex polysaccharide of the primary cell wall. AT2G45220.1 [77] catalyzed the homogalacturonan backbone, remodeling the cell wall and modifying the physicochemical properties of pectin. AT1G05675.1 [78] was involved in pectin biosynthesis as a glycosyltransferase. AT2G45220.1 and AT1G05675.1 were homologous to Glyma.03G029000 and Glyma.19G025100 on chromosomes 3 and 19, respectively. Xylan is a polysaccharide mainly found in the secondary cell wall (SCW). AT1G27930.1 [79] modified xylan in the SCW by catalyzing the 4-O-methylation of glucuronoxylan. AT1G27930.1 was homologous to Glyma.11G251000 on chromosome 11.

Some *A*. *thaliana* genes were associated with signal transduction. It is well-recognized that SCW-associated NAC domain proteins are TFs and control SCW biosynthesis as central genes in a regulatory gene network [80–81]. AT4G29230.1 [82] regulated SCW biosynthesis by activating SCW NAC binding elements and initiating the transcription of target genes. AT4G29230.1 was homologous to Glyma.05G108700 on chromosome 5. Four MYB TF paralogs, AT2G38300.1, AT2G32460.1, AT3G09370.1, and AT5G15310.1, affected the transcriptional regulation of SCW biosynthesis [83]. AT2G38300.1, AT2G32460.1, AT3G09370.1, and AT5G15310.1 were homologous to Glyma.02G282900, Glyma.12G193300, Glyma.16G137000, and Glyma.19G024700 on chromosomes 2, 12, 16, and 19, respectively. A leucine-rich repeat receptor-like kinase (LRR-RLK) is a receptor on the plant cell membrane, receiving external signals for intracellular signal transduction. Four LRR-RLK families, AT1G49490.1, AT3G19020.1, AT2G33170.1, and AT2G36570.1, functioned as regulators of SCW formation [84]. AT1G49490.1, AT3G19020.1, AT2G33170.1, and AT2G36570.1 were homologous to Glyma.07G164300, Glyma.10G090800, Glyma.13G227800, and Glyma.19G190200 on chromosomes 7, 10, 13, and 19, respectively.

According to QTL information from previous soybean population studies, fifteen out of 86 QTLs for pod dehiscence exhibited pleiotropic effects on various agronomic traits, including the first flower, pod maturity, seed composition, seed weight, and yield [85–102] (S9 Table). Eight out of 15 QTLs for pod dehiscence were associated with seed weight or yield, implying that pod dehiscence significantly impacted soybean yield (S9 Table). Many studies have reported the relationship among agronomic traits in soybean. As major agronomic traits, the first flower, pod maturity, seed weight, and yield were coinherited with pod dehiscence [9,22,23]. It was well-known that seed weight positively correlated with yield [103–104]. The genotypic correlation between yield and oil was positive, and the genotypic correlation between oil (and yield) and protein was negative [105–107].

From the perspective of soybean production, we need to consider two key points to reduce pod dehiscence. First, monitoring environmental conditions during the harvest period is important. After R8 (full maturity stage), soybean harvesting typically requires 5 to 10 days of dry weather to reduce the seed moisture level to 13% [11]. Pod dehiscence-susceptible soybean cultivars resulted in yield losses of 40–100% under severe dry weather conditions or harvesting delays caused by frequent rainfall [25–26]. Climate data analysis showed that the Southern region of the USA has experienced significantly higher temperature and precipitation than the Midwest over the past 125 years (S1 Fig). The subtropical climate of the Southern USA has created suitable conditions for accelerating pod dehiscence [9,13,25]. In the Southeastern USA, pod dehiscence contributed 37% (53–310 kg ha^-1^) of total soybean field loss, and the early harvesting system mitigated this loss [24]. Therefore, effective field management plans will be required to prevent huge yield loss from pod dehiscence.

Second, it is critical to develop pod dehiscence-resistant cultivars with the optimal haplotype using MAS and GS. We identified accessions with the best haplotype and the top 5 SNPs with the highest selection efficiency in MAS. Because of differences in population structure among *G*. *max* populations (S4 Fig), it was challenging to determine the consensus favorable allele of each QTL across the *G*. *max* populations (S7 Table). Nevertheless, information about 86 QTLs (S4 Table) and the top 5 SNPs (Table 2) would be valuable for MAS in further population studies.

Previously reported QTLs were used to confirm 12 out of 86 QTLs (S4 Table). *NST1A* was not located within 95% CI of the QTL position for ss715598070. However, *NST1A* was closely linked to ss715598144 within a 4 kb range, and the LD between ss715598070 and ss715598144 was considerably high across the *G*. *max* populations (average *r*^2^ = 0.71). ss715598070 and *NST1A* are likely to be inherited together. One RFLP [10] and eight SSRs [15–16] (S5 Table) were identified in three RIL populations. The RIL populations exhibited a small population size ( < 200). The Beavis effect [108] may decrease R^2^ values and overestimate 95% CIs in the function [64]. Additionally, a small number of markers were used to construct genetic maps of the RIL populations, implying that a lower marker density may decrease R^2^ values and impair QTL detection power [109]. Therefore, except for ss715623567 and ss715624201, the rest of the 10 QTLs will require validation in future studies.

In the comparison between MAS and GS in 1IL64, MAS outperformed GS, showing higher selection efficiency. PI89059 was the accession with the best haplotype in MAS. However, the accession was positioned in the middle of the GBV distribution in sBLUP (Fig 6). These different results between MAS and GS appeared to be due to the GS model. sBLUP utilized the additive genetic effects of all polymorphic SNPs across the genome [67]. The accessions selected by MAS and GS in this study would be useful sources for determining the optimal haplotype of pod dehiscence-resistant cultivars. It will be essential to acquire more population data for MAS by confirming additional QTLs, assessing QTL stability, and testing QTL gene action in future population studies. On the other hand, assessing other advanced GS models will be necessary to find the best-fit model.

## Conclusion

Pod dehiscence is a well-known trait with high heritability in soybean, although some climate factors, such as temperature and precipitation, significantly impact pod dehiscence. As a domestication trait, pod dehiscence is a primary cause of yield loss in cultivated soybean. We obtained data for one *G*. *soja* and four *G*. *max* populations from the USDA database. GWAS identified 86 QTLs using four *G*. *max* populations. Eight out of 86 QTLs were related to the domestication of pod dehiscence. MAS and GS were implemented to select pod dehiscence-resistant accessions with the best haplotype and lowest GBV. The selected accessions would assist in identifying the optimal haplotype for developing pod dehiscence-resistant cultivars. However, further studies will be required to obtain additional QTLs and evaluate advanced GS models.

## Supporting information

S1 Fig Statewide average temperature and precipitation from September to November, spanning the years 1895-2020 in the USA. Red dots showed the field locations of four *G. max* populations. The adaptation zones for soybean MGs and four divisions in the USA were displayed on the left and right climate maps. Both climate maps were obtained from the NCEI.
(PPTX)

S2 Fig Putative domestication-related SNPs with significantly different allele frequencies between WS1179 and a G. max population. A total of 2,204 SNPs (magenta vertical lines) were significant in all *G. max* populations. From the rest of the 2,076 SNPs, 284 SNPs (dark orange vertical lines), 173 SNPs (golden brown vertical lines), 292 SNPs (green vertical lines), and 1,327 (blue vertical lines) were significant in 1IL64, 1IL66, MN0102, and MS967, respectively.
(PPTX)

S3 Fig GO analysis of 1,515 putative domesticated-related genes. The pie chart displayed the percentage of annotated gene numbers for each GO term. The biological process GO terms were presented on the right.
(PPTX)

S4 Fig Population structure in WS1179, 1IL64, 1IL66, MN0102, and MS967. In the individual population structure image, a single vertical line corresponded to an accession. The single vertical line was divided into K-colored segments, and the segment length was proportional to each K-inferred cluster. The numbers below the individual population structure image indicated the number of subpopulations, K.
(PPTX)

S5 Fig QTL detected by multiple GWAS models in four G. max populations. The circular (top) and quantile-quantile (bottom) plots of 1IL64, 1IL66, MN0102, and MS967 were arranged from left to right. Only the circular and Q-Q plots for a combination of PC and marker-based *k* matrices were presented. In the circular plot, a red asterisk showed the position of a QTL, and a LOD value was used instead of a *q*-value (*q*-value = 10^-LOD). A red dotted circle in the circular plot indicated the threshold for QTL detection in each GWAS model.
(PPTX)

S1 Table Information about twenty-four *G*. *max* populations for pod dehiscence study in the GRIN. (XLSX)

S2 Table Information about 1,179 wild accessions in WS1179. (XLSX)

S3 Table *P*-values for *χ* ^2^ goodness of fit tests and F_ST_ values in 4,280 SNPs between WS1179 and a *G*. *max* population. (XLSX)

S4 Table Information about 86 QTLs identified in four *G*. *max* populations. (XLSX)

S5 Table QTL information of one RFLP and eight SSRs from three RIL population studies. All RIL population studies provided limited information about the CIs of QTL positions. The 95% CIs of QTL positions were estimated using the following equation: 95% CI for a RIL population = 163 / (N ×  R^2^) (R^2^: phenotypic variance explained by a QTL and N: population size) [64]. The positions and 95% CIs of all QTLs were projected onto the Consensus 4.0 genetic map for QTL confirmation. Therefore, left and right flanking makers covering 95% CIs were determined based on the Consensus 4.0 genetic map. NA stands for “not available.”
(XLSX)

S6 Table Soybean candidate genes from 86 QTLs for pod dehiscence. *A*. *thaliana* genes homologous to soybean candidate genes were included in the table. No soybean candidate genes for 8 QTLs on chromosomes 4, 7, 8, 13, 17, and 20 were found. NA stands for “not available.”
(XLSX)

S7 Table Favorable alleles of 86 QTLs in each *G*. *max* population. (XLSX)

S8 Table GBV and PEV for each accession in 1IL64 using sBLUP. (XLSX)

S9 Table Different agronomic traits associated with pod dehiscence. From 86 QTLs for pod dehiscence, a 10 kb window of each QTL was used to find QTLs for different agronomic traits based on previous soybean population studies. Favorable alleles contributed to the decrease in the number of open pods at harvest.
(XLSX)
