# Supplementary figures and images for: Genome-wide association and selection studies for pod dehiscence resistance in the USDA soybean germplasm collection
Source: PLoS One. 2025 Mar 28;20(3):e0318815. doi: 10.1371/journal.pone.0318815 (PMC11952757; doi:10.1371/journal.pone.0318815)

## Slide 1
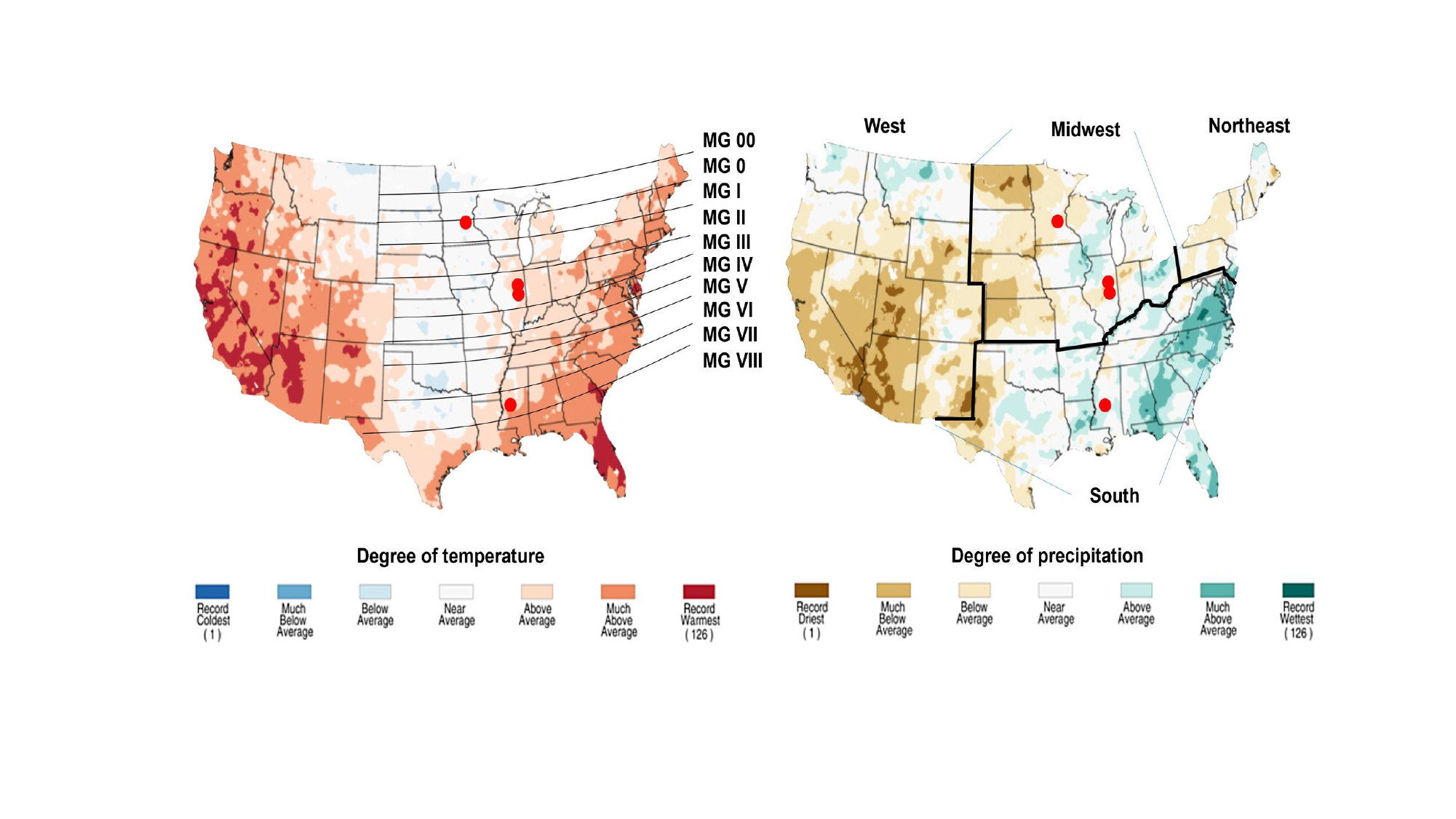

Supplement: S1 Fig — Red dots showed the field locations of four G. max populations. The adaptation zones for soybean MGs and four divisions in the USA were displayed on the left and right climate maps. Both climate maps were obtained from the NCEI. (PPTX) [file pone.0318815.s001.pptx]

## Slide 1
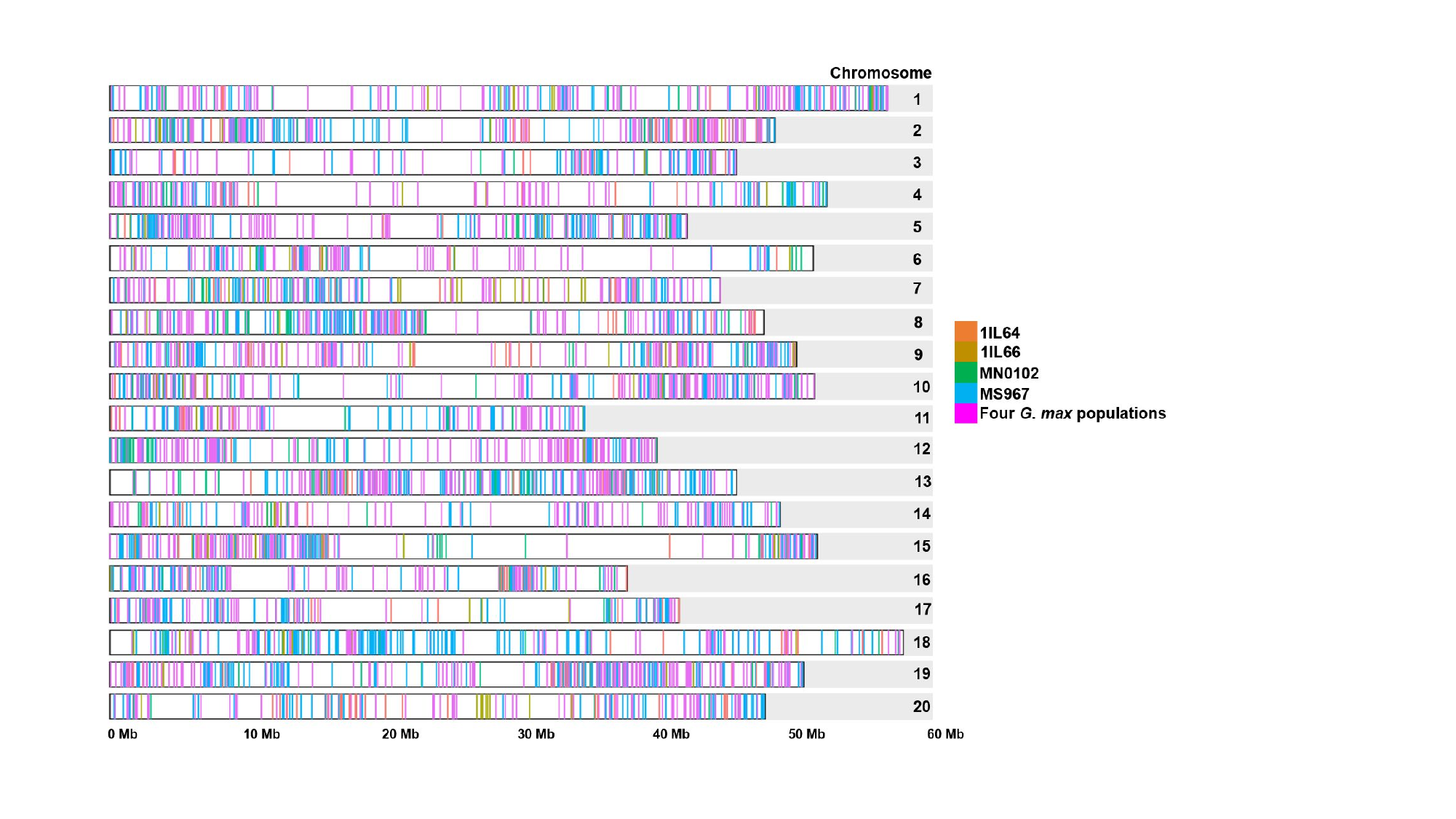

Supplement: S2 Fig — Putative domestication-related SNPs with significantly different allele frequencies between WS1179 and a G. max population. A total of 2,204 SNPs (magenta vertical lines) were significant in all G. max populations. From the rest of the 2,076 SNPs, 284 SNPs (dark orange vertical lines), 173 SNPs (golden brown vertical lines), 292 SNPs (green vertical lines), and 1,327 (blue vertical lines) were significant in 1IL64, 1IL66, MN0102, and MS967, respectively. (PPTX) [file pone.0318815.s002.pptx]

## Slide 1
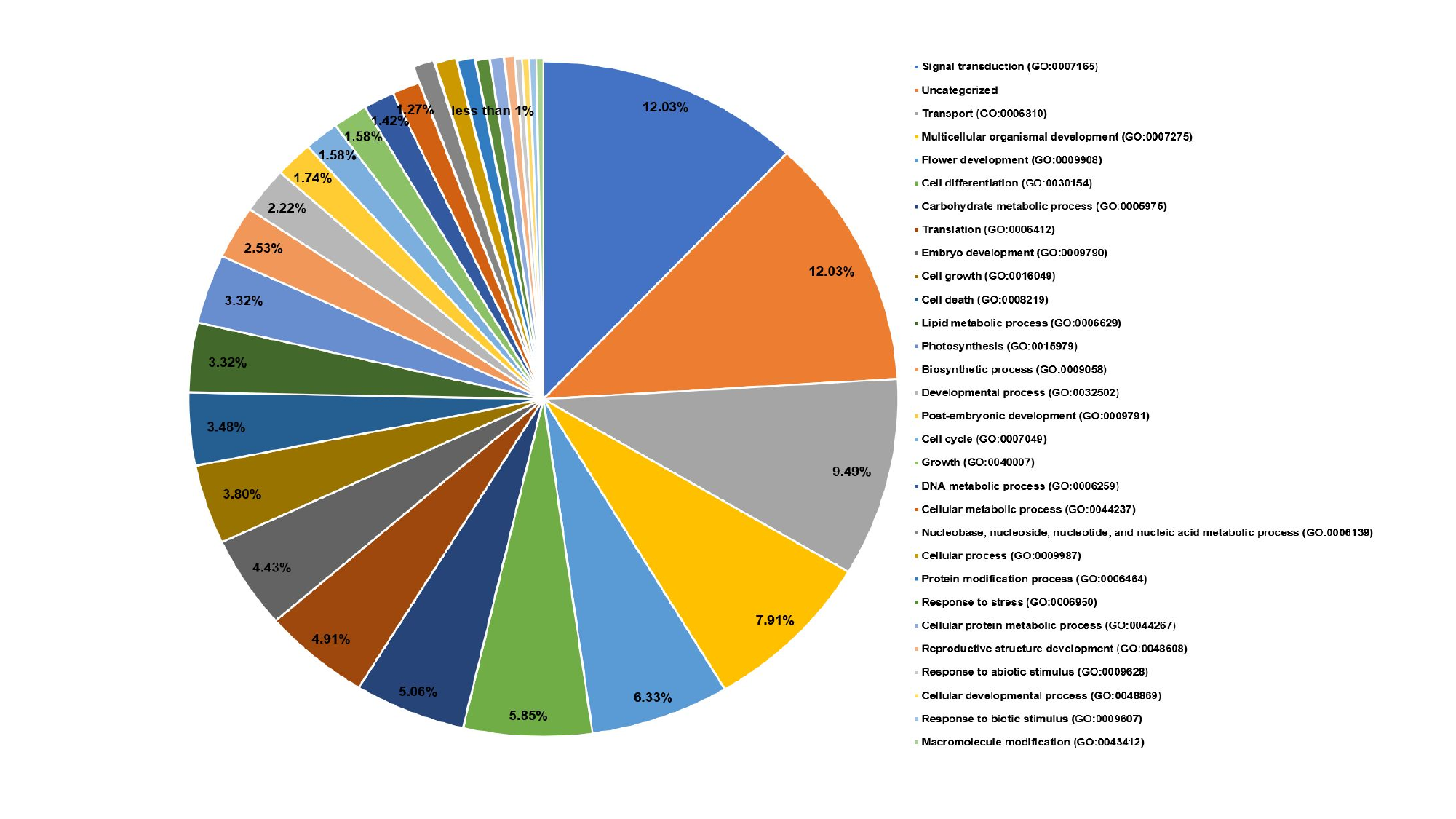

Supplement: S3 Fig — The pie chart displayed the percentage of annotated gene numbers for each GO term. The biological process GO terms were presented on the right. (PPTX) [file pone.0318815.s003.pptx]

## Slide 1
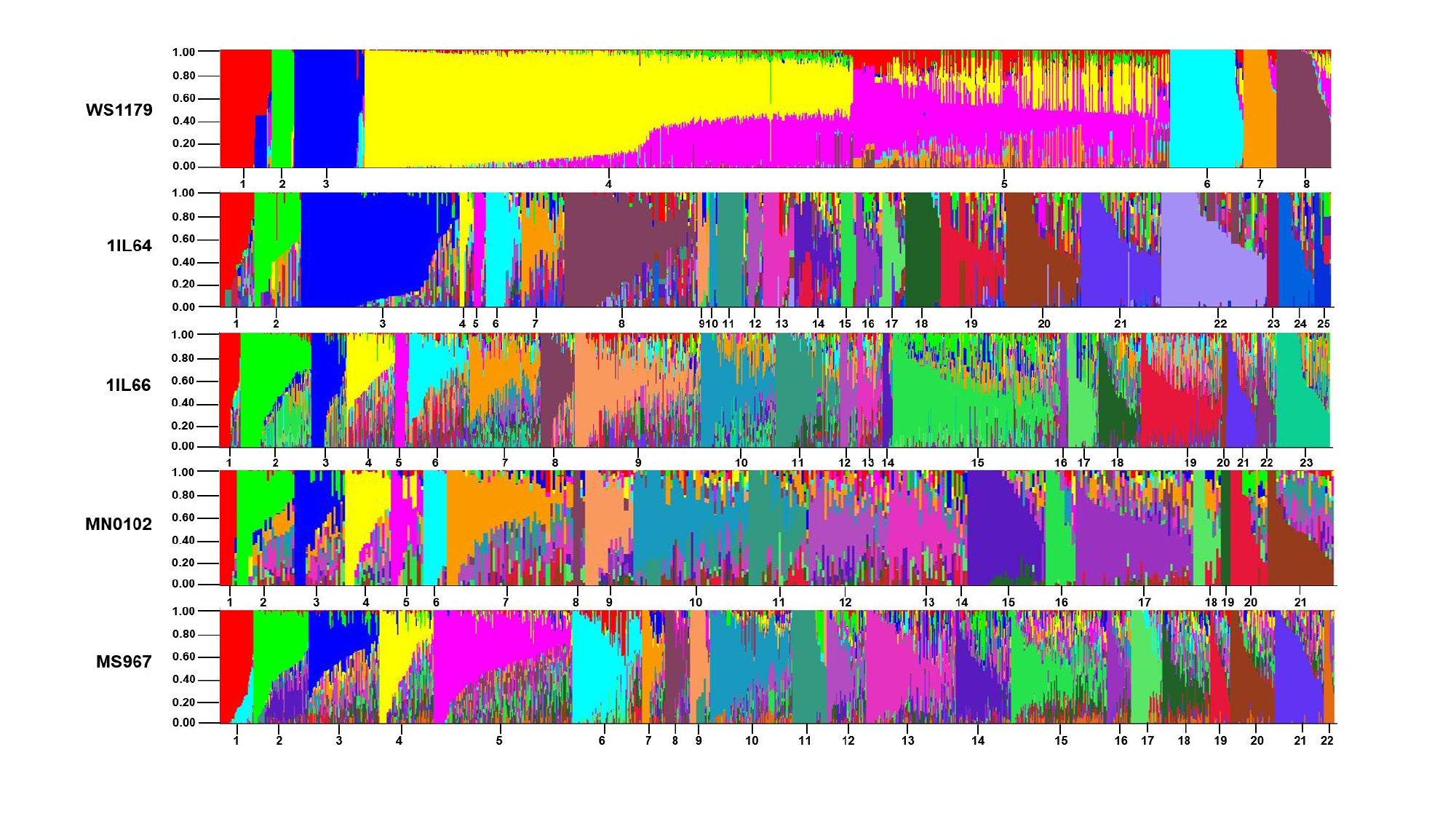

Supplement: S4 Fig — In the individual population structure image, a single vertical line corresponded to an accession. The single vertical line was divided into K-colored segments, and the segment length was proportional to each K-inferred cluster. The numbers below the individual population structure image indicated the number of subpopulations, K. (PPTX) [file pone.0318815.s004.pptx]

## Slide 1
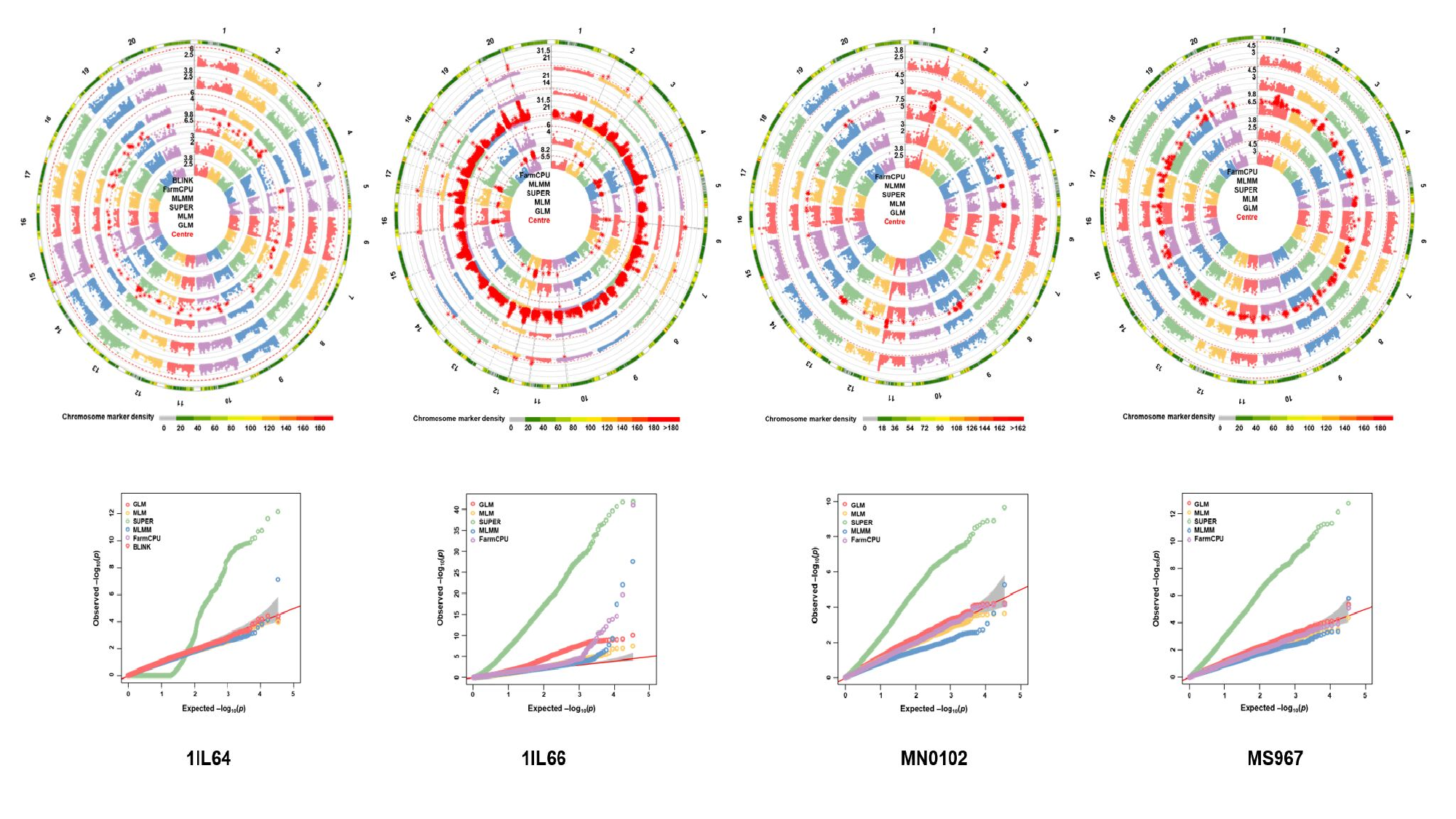

Supplement: S5 Fig — The circular (top) and quantile-quantile (bottom) plots of 1IL64, 1IL66, MN0102, and MS967 were arranged from left to right. Only the circular and Q-Q plots for a combination of PC and marker-based k matrices were presented. In the circular plot, a red asterisk showed the position of a QTL, and a LOD value was used instead of a q-value (q-value = 10^-LOD). A red dotted circle in the circular plot indicated the threshold for QTL detection in each GWAS model. (PPTX) [file pone.0318815.s005.pptx]
